# Effects of Nitrogen Addition on Plant Properties and Microbiomes Under High Phosphorus Addition Level in the Alpine Steppe

**DOI:** 10.3389/fpls.2022.894365

**Published:** 2022-06-20

**Authors:** Junfu Dong, Xiaoyong Cui, Haishan Niu, Jing Zhang, Chuanlu Zhu, Linfeng Li, Zhe Pang, Shiping Wang

**Affiliations:** ^1^Institute of Marine Science and Technology, Shandong University, Qingdao, China; ^2^College of Life Sciences, University of Chinese Academy of Sciences, Beijing, China; ^3^College of Resource and Environment, University of Chinese Academy of Sciences, Beijing, China; ^4^College of Grassland Science, Beijing Forestry University, Beijing, China; ^5^Institute of Tibetan Plateau Research, Chinese Academy of Sciences, Beijing, China

**Keywords:** the core species, the Qinghai-Tibetan plateau, nutrient uptake, plant–microbe interaction, nitrogen application

## Abstract

Nitrogen (N) addition can increase the vegetative growth, improve the plant production, and restore the degraded terrestrial ecosystems. But, it simultaneously aggravates the soil phosphorus (P) limitation for plant growth, thus affecting its positive effects on ecosystems. However, how plants and soil microorganisms will change under conditions of high P content in soil is still unknown. In this study, we explored the effects of three levels of N addition (0, 7.5, and 15 g.N.m^–2^.year^–1^) on plants and microorganisms at the high P addition level (13.09 g.P.m^–2^.year^–1^) in the alpine steppe. We found that the soil microbial community composition had no significant difference between different N addition levels, and the soil AN and AP had a significant effect on the phospholipid fatty acid (PLFA) composition. The abundance of the core PLFAs (i.e., 16:1ω7c, 16:0, a17:1, i17:0, 18:1ω9c, and 18:1ω7c) also remained unchanged after N addition, and microbes at individual, population, and community levels were all correlated with SOM, AK, AN, and pH. Conversely, plant biomass and nutrient content showed linear trends with increasing N addition, especially the dominant functional groups. Specifically, the biomass and plant tissue N content of *Gramineae*, and the total N content of aboveground biomass were all improved by N addition. They were correlated with soil ammonium and AP. The structural equation modeling (SEM) demonstrated that N addition had a direct negative effect on soil microbial biomass, but an indirect positive effect on aboveground biomass *via* soil ammonium. These findings clarify the importance of N-amendment in regulating plants and microorganisms under high P conditions and provide a better understanding of the N-added effects in the alpine steppe.

## Introduction

Nitrogen (N) is an essential macro-element for plant growth and development (Mu and Chen, 2021), and they were usually transported to terrestrial ecosystems by anthropogenic N input and natural N deposition (Han et al., 2020). Most previous studies were mainly focused on the responses of plant biomass (Fu and Shen, 2016; Chen et al., 2018) and plant diversity (Foster and Gross, 1998; Bird and Choi, 2017; Soons et al., 2017) to N addition in different terrestrial ecosystems. With the increasing N addition, some studies revealed that the plant diversity was reduced (Bobbink et al., 1998; Stevens et al., 2004; Bird and Choi, 2017; Luo et al., 2019), while others were increased below 8.7 and 13.4 kg N ha^–1^.year^–1^ in open and closed-canopy vegetation across the continental United States (Simkin et al., 2016). Moreover, some results indicated that the plant diversity and biomass had no response after N addition into the tropical forest and alpine steppe, respectively (Lu et al., 2010; Dong et al., 2016). In addition, by collecting six local plant species (including *Erythronium Americanum*, *Dryopteris intermedia*, *Oxalis acetosella*, *Acer saccharum*, *Viola macloskeyi* F. Lloyd, and *Viola macloskeyi*) in a second-growth northern hardwood forest within the Catskill State Park in New York, Tessier and Raynal (2003) found that the concentrations of plant N were significantly different between plant species with *Oxalis* and *Viola* having the highest and Acer having the lowest, but not for plant populations at varied N-addition levels. Some studies contributed these controversies to the competitiveness of specific plant species that prefer higher N conditions or to eutrophication and soil acidification (Stevens et al., 2018) and the rate and period of N addition (Lu et al., 2010; Dong et al., 2016). Therefore, a better understanding of how plants respond to N addition is critical for maintaining biodiversity and improving plant production.

The impacts of N addition on plants were usually not only by affecting N element but also by interacting with phosphorus (P) to influence the N-induced impacts by creating a N:P imbalance in terrestrial ecosystems (Vitousek et al., 2010; Peñuelas et al., 2013). Before industrial revolution, plants mainly absorb P from soil parent materials, thereafter fertilizers become an essential source (Cordell et al., 2009; Vitousek et al., 2010; Elser and Bennett, 2011), resulting in substantial transfers of P in different ecosystems (Peñuelas et al., 2013). In some terrestrial ecosystems, such as forest, steppe, and meadow, combined application of N and P can enhance plant N and P uptake (Lü et al., 2013, 2016), while the sole application could cause N:P imbalance for plants (Peñuelas et al., 2013) and the aboveground biomass showed an asymptotic relationship with changes of the tissue N:P ratio (Peng et al., 2019). In addition, the impacts of N addition on soil microorganisms were mitigated by P addition in a P-limited paddy soil (Su et al., 2015), which changed their interaction with plants. These findings not only confirmed the positive effects of N addition on plant production but also highlighted that the un-continued positive effects were always related to the soil P conditions (Bobbink et al., 2010; Chen et al., 2019). However, it is still unknown whether P limitation is the main factor for the continued positive effects of N addition on terrestrial ecosystems.

Soil microbes play critical roles in global biogeochemical cycling and form strong bonds with plants in ecosystems (Van Der Heijden et al., 2008). Previous findings indicated that soil microbes can promote the plant growth by enhancing their nutrient acquisition (Adesemoye et al., 2009; Richardson et al., 2009). Under some scenarios in terrestrial ecosystems, soil microbes even act as drivers to plant community structures (Van Der Heijden et al., 2008). Moreover, soil microbes mediated the bioavailability of soil nutrients and aggregation formation (Rashid et al., 2016); conversely, soil microbes were also affected by N addition (Treseder, 2008; Leff et al., 2015; Zeng et al., 2016; Luo et al., 2019). However, some studies revealed that the microbial biomass could remain stable after N addition in the hardwood and pine stands (Frey et al., 2004). After N and P addition, the balance of soil N:P was disturbed, and soil archaea and bacteria responded differently to N, P, and NP additions due to their various urgent needs for N, P, or other resources (Adomako et al., 2022). Furthermore, soil microbes can alter the effects of N:P balance on plant performance, which also depends on nutrient conditions (Ma B. et al., 2019). Based on their key roles in the ecosystem, soil microbes had strong correlations with plants, anyway. Recent studies found that N addition may mediate edaphic properties firstly (Hu et al., 2010; Kang et al., 2018), and then changed the microbial community (Sarathchandra et al., 2001; Eghball, 2002). As the sensitive indicators of surrounding disturbances (Ma X. et al., 2019; Xiao et al., 2019), the soil microbial biomass and community structure, plants biomass, and stoichiometry can respond immediately to N addition which will help us to evaluate the N:P balance and manage the ecosystems, which need further exploration.

Given these problems, we conducted N-added field experiments in the alpine steppe, and previous studies proved that the alpine steppe is sensitive to climate change (Liu et al., 2013), especially in the Tibetan Plateau, which is more vulnerable and promptly responds to climate changes compared to most other regions on Earth due to its ecological fragility (Zhong et al., 2019). It was also reported that the annual N deposition rate reached 15.2 kg N ha^–1^ from 2010 to 2014 in this region (Xu et al., 2015), and prediction showed that the rate will be twice higher than that in the early 1990s by 2050 (Galloway et al., 2004; Basto et al., 2015). In addition, our previous studies found that P is a limited factor for plant and soil microbes at the same field station (Dong et al., 2016, 2020b). These phenomena would be an enormous disaster for the ecosystem, while we still do not know (1) how the plants and soil microbes will change, and (2) whether the positive effects of N addition on them will be continued at high P addition levels in the alpine steppe. Combined with N addition, we also added P fertilization to create a higher soil P condition, and we infer that the P is the main limited factor for plants and microbes if their biomass or nutrient properties would increase linearly with increasing N fertilization, otherwise the soil P is not the main limited factor for the ecosystem. These explorations will be helpful for humans to understand the impacts of increasing N content under conditions of high P levels, and this might help us to better understand the N-added effects on terrestrial ecosystems.

## Materials and Methods

### Introduction of the Field and Experimental Design

The field experiment (N31°26′, E90°02′, 4678 m a.s.l.) was performed in Baingoin County, Tibet Autonomous Region in southwest China (Supplementary Figure 1). This area is a semiarid cold alpine steppe and the soil is Gelic Cambisols according to the Food and Agriculture Organization of the United Nations (FAO) (Baumann et al., 2009; Dong et al., 2020a). As mentioned in our previous articles (Dong et al., 2016, 2020a), *Stipa purpurea* is the dominant plant species, and the accessory plant species are *Leontopodium leontopodioide* and *Heteropappus bowerii* in this place. The average annual precipitation is 301.2 mm, of which 80% falls in the growing season from June to September. The mean annual temperature is −1.2°C, and the maximum mean monthly temperature is 14.7°C in July. The background information of soil properties is presented in Table 1, which was also described in our former publication (Dong et al., 2016). To introduce it briefly, the soil TC, TN, and TP were 32.53, 1.65, and 0.62 g/kg, respectively; the soil AN and AP were about 128 and 5 mg/kg period for the establishment of treatments, and the soil pH was nearly 7. In addition, our experimental plots were grazed daily by yaks and sheep before fencing, and no fertilizing history was found. Due to overgrazing, they have been moderately or severely degraded.

**TABLE 1:** The background information of soil properties before the experiment (Dong et al., 2016).

	SOM (g/Kg)	TN (g/Kg)	TP (g/Kg)	AN (mg/Kg)	AP (mg/Kg)	pH
0–10 cm	32.53	1.65	0.62	128.17	4.96	6.97
10–20 cm	18.8	1.09	0.74	77.3	3.04	7.04

*SOM indicates total organic matter content in soil, TN indicates total nitrogen content in soil, TP indicates total phosphorus content in soil, AN indicates available nitrogen content in soil, and AP indicates available phosphorus content in soil.*

The experimental plots were conducted by completely randomized block design in July 2013 on this field station. In each of the five blocks, three subplots were randomly assigned to three N additions (0, 7.5, and 15 g N m^–2^ year^–1^, applied as urea), and each subplot was simultaneously fertilized with high P addition (13.09 g P m^–2^ year^–1^, applied as monocalcium phosphate) to create a higher P condition. Each subplot was 5 × 5 m with five duplications and a 2-m buffer zone of any adjacent plots (Supplementary Figure 1). The dry powder of fertilizers was eventually applied over the respective plot at dusk twice each year at the time of the beginning and the vigorous period of plant growth.

### Sampling and Analyses

At the vigorous period after 30 days of the second fertilization in September 2014, we surveyed the plant community (i.e., the height, coverage, and plant species) in a 1 × 1 m quadrate of each subplot. Briefly, the quadrates were randomly established in each subplot of four blocks, and each quadrat was divided into equal 100 small subquadrates (1 × 1 cm). We measured the plant height and recorded plant species at the same vertex of each subquadrate. After surveying the plant community, the aboveground biomass was clipped at the ground level and sorted by plant species at the same quadrate. Then, we collected the topsoil layer (0–10 cm) samples by mixing seven soil cores (3.5 cm diameter) in the same clipped subplots. Then, the plant samples were dried at 65°C until constant weight; soil samples were preprocessed to pick out the visible roots and stones and sieved through a 2-mm mesh, and finally separated into two subsamples. A subsample was stored in the room after being air-dried at room temperature for the analysis of some soil physicochemical properties, and the other subsample was stored in a refrigerator at −80°C for soil microbial analysis. The contents of soil organic matter (SOM), soil total N (TN), soil total P (TP), soil available N (AN), soil available P (AP), available potassium (AK), and soil pH were determined using the air-dried soil; the content of soil NH_4_^+^-N and moisture content (SMC) were determined using the fresh soil (Dong et al., 2016, 2020a).

Soil organic matter was measured using potassium dichromate oxidation and back titration with ferrous sulfate. SMC was determined by a gravimetric method after drying at 105°C for 24 h. AN was determined by the alkaline hydrolysis method. AP was determined using the molybdenum blue method after being extracted with sodium bicarbonate from soil samples. AK was determined using a flame photometric method after being extracted with ammonium acetate (Bao, 2000). Soil pH was measured by using a pH meter (OAKTON^®^ pH, Oakton Instruments, Vernon Hills, IL, United States) at a ratio of 1:5 (weight/volume) for soil vs. distilled water. The content of soil NH_4_^+^-N was measured using an autoanalyzer (SmartChem140, AMS Alliance, Guidonia, Italy) in 2 M KCl extracts (1:4, soil: extractant). The dried soil samples were ground to a fine powder (through 0.15 mm sieve) to measure the TN and TP using the Kjeldahl method (Liao, 1981) and the molybdenum blue method with an ultraviolet-visible spectrophotometer (UV-2700, Shimadzu, Kyoto, Japan), respectively. The plant samples of each functional group (*Gramineae: S. purpurea, Poaannual*, and *Festuca coelestis; Compositae: L. leontopodioide* and *Heteropappus Puppyflower; Cyperaceae: C. oxyleuca V. Krecz, Carex moorcroftii*, and *Kobresia pygmaea*; and forb for other plants) were ground to a fine powder (using a 0.15 mm sieve) by mixing plant aboveground biomass according to their relative biomass occupied by the whole functional group, and then the total N and total P of each plant functional group were determined by using indophenol blue colorimetry and the Mo-Sb colorimetric method after being digested with H_2_O_2_-H_2_SO_4_, respectively (Dong et al., 2020b).

Phospholipid fatty acid (PLFA) profiling has a confidential ability to quantify the responses of soil microbes (Orwin et al., 2018). We used the standard procedure to extract PLFAs from 10 g of fresh soil, as described in detail by Frostegård and Bååth (1996). Briefly, soil samples were extracted using an extraction mixture of chloroform:methanol:phosphate buffer (1:2:0.8, v/v/v). The extracted fatty acids were then fractionated using solid-phase extraction columns with chloroform, acetone, and methanol, respectively. Phospholipids were trans-esterified to fatty acid methyl esters (FAMEs) with 1:1 methanol:toluene and 0.2 M potassium hydroxide. Methyl nonadecanoate (19:0) was used as an internal standard to calculate each individual fatty acid concentration. The FAMEs were identified by using the MIDI Sherlock Microbial Identification System 6.0 (Microbial ID, Inc., Newark, DE 19713.) The abundance of individual PLFAs was expressed as nmol PLFA g^–1^ dry soil. We found that 22 biomarkers appeared in almost all of samples in this study. The gram-positive bacteria (G+) were presented by i15:0, a15:0, i16:0, i17:0, and a17:0; 16:1ω7c, cy17:0, 18:1ω7c, and cy19:0 were used to present the gram-negative bacteria (G−); the total soil bacteria were presented by combining G+ and G−. The saprotrophic fungus was presented by 18:1ω9c and 18:2ω6,9c; 16:1ω5c was used to present the arbuscular mycorrhizal fungus (AMF), and the total biomarkers of saprotrophic fungus and AMF were used to present the fungus. Notably, 16:0 10-methyl and 18:0 10-methyl were used to present the actinomycetes. Except bacteria, fungus, and actinomycetes, the total microbes were also present by the combination of i15:1, i16:1, 16:0 N alcohol, 16:0, a17:1, 17:1ω8c, 18:1ω5c, and 18:0 (Dong et al., 2020b).

### Statistical Method

The responses of soil microbial community and plant properties to N addition at a higher P level were revealed using non-metric multidimensional scaling (NMDS) and permutation multivariate analysis of variance (PERMANOVA) using the adonis function in R package vegan. These analyses were performed by using individual PLFAs to reveal soil microbial community and by using plant traits (plant biomass and nutrient properties of all functional groups) to reveal plant community, respectively. The principal component analysis (PCA) was used to reduce the dimension and find the core factors based on their explained contribution to the first two dimensions (Zosso and Wiesenberg, 2021). The main effects of N addition on edaphic properties, plant properties, and soil microbes were analyzed by one-way analyses of variance (ANOVA) followed by a *post-hoc* mean test (LSD). Redundancy analysis (RDA) was applied to explore a combination of soil physicochemical properties that could explain the divergence in soil microbes and the plant community structure. The Pearson’s correlations between soil physicochemical properties and plant properties or soil microbes were also calculated. The structural equation modeling (SEM) was used to explore the relationships between soil microbes, plant biomass, plant-nutrient traits, and edaphic properties by using the AMOS software (IBM SPSS AMOS 25, Chicago, IL, United States). All analyses were conducted using the R software v3.4.4.^1^ The histograms and scatterplots were created using OriginPro 2017 (OriginLab Corporation, Northampton, MA, United States).

## Results

### Community Responses of Soil Microbes and Plants

**FIGURE 1:**
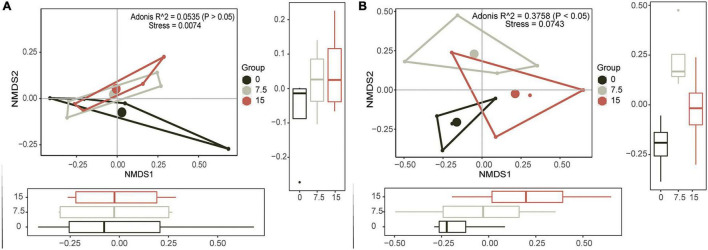
Nonmetric multidimensional scaling (NMDS) plots show the relative differences in community composition of soil microbes **(A)** and plant **(B)** along the increasing N gradient.

To identify the principal PLFAs that caused the changes of microbial community, we employed PCAs based on all identified PLFAs. Principal components (PC) 1 (explained 68.9%) and PC2 (explained 27.4%) explained 96.3% of the variances (Supplementary Figure 2), and they were illustrated by six individual PLFAs (16:1ω7c, 16:0, a17:1, i17:0, 18:1ω9c, and 18:1ω7c), which were named core microbes (Supplementary Figure 2). Furthermore, the NMDS results showed that soil microbial community had no significant responses at different N application rates, and there was no linear trend with increasing N addition (Figure 1A).

Similar to soil microbial community, PC1 (explained 85.4%) and PC2 (explained 12.2%) together explained most variances (Supplementary Figure 2), and six plant variables (i.e., biomass of *Gramineae*, biomass of *Compositae*, and the total aboveground biomass, and they were termed as Plant_biomass; plant tissue N content of *Gramineae*, plant tissue content N of *Compositae*, and total plant tissue N contents, and they were termed as Plant_nutrient) were selected as the core plants traits (Supplementary Figure 2). Results of NMDS and PERMANOVA of plants showed that there were significant differences between varied N application rates, and there was a linearly changed trend, especially along the NMDS1 orientation (Figure 1B).

### Responses of Core Microbes and Plant Traits

**FIGURE 2:**
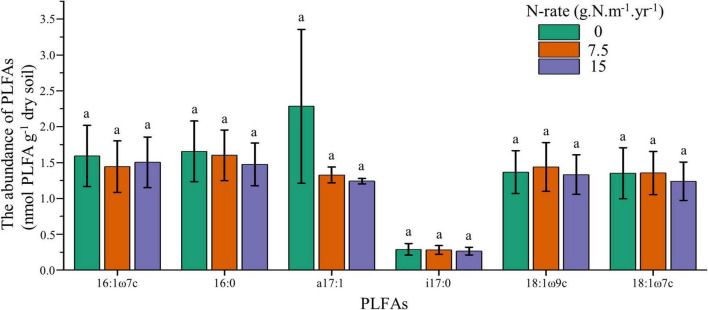
Changes of core microbes under different N application rates. Treatments are expressed by data with means ± SE (*n* = 4). Different letters above boxes indicate significant differences between N application rates at the *P* < 0.05 level.

To further explore the responses of individual PLFAs upon increasing N addition, we employed one-way ANOVA for core microbes. Results showed that the core microbes had no significant difference between N application rates, and these individual PLFAs also had no linear trend with increasing N fertilization (Figure 2). These results confirmed that soil microbes had no responses to N addition, both at individual and community levels.

**FIGURE 3:**
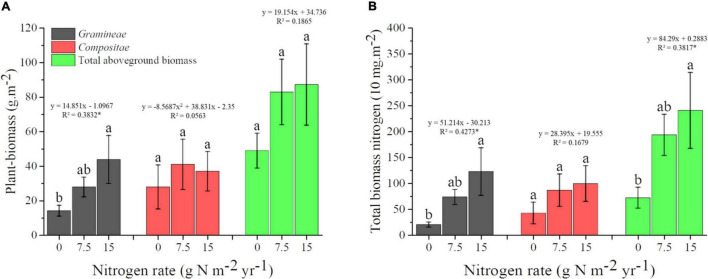
Changes of plant-biomass **(A)** and total biomass nitrogen **(B)** under different N application rate. Treatments are expressed by data with means ± SE (*n* = 4). Different letters above boxes indicate significant differences between N application rates at the *P* < 0.05. Results of regression analysis were shown above boxes using its function and R2. The * indicates there were significant correlations between plant traits and N application rates at the *P* < 0.05 level.

For Plant_biomass, results of one-way ANOVA showed that the biomass of *Gramineae* was increased by N addition, and each 1 g N m^–2^.year^–1^ shift was associated with a 14.851 g.m^–2^ aboveground biomass change. Moreover, there was a linear trend for *Gramineae* biomass with increasing N fertilization, while there was no significant linear trend for the biomass of *Compositea* and the total aboveground plant (Figure 3A). For Plant-nutrient, our results indicated that the TN of *Gramineae* and total plant community were all increased by N addition, and they all showed a linear trend with increasing N fertilization. In addition, each 1 g N m^–2^.year^–1^ shift was associated with 512.14 and 842.9 mg.m^–2^ TN for *Gramineae* and total plant community, respectively (Figure 3B).

### Edaphic Factors Controlling Plants and Microbes

**FIGURE 4:**
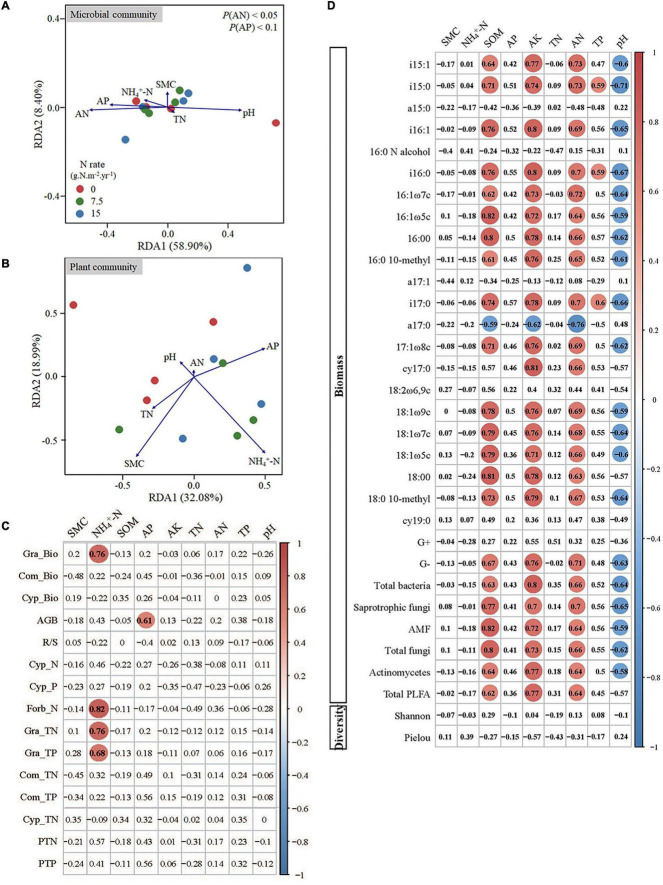
Redundancy analysis biplot for the correlation of plant **(A)** and microbial community **(B)** with soil properties, and the correlations between soil properties and plant traits **(C)** and microbes **(D)**. Red circles in **(C)** and **(D)** indicate significant positive correlations, and blue circles indicate significant negative correlations. SMC indicates the soil moisture content; SOM indicates the soil organic matter content; AP indicates the soil available P; AK indicates the soil available potassium; TN indicates the soil total N content; AN indicates the soil available N; TP indicates the soil total P content; Gra indicates Gramineae; Com indicates Compositae; Cyp indicates Cypositae; AGB indicates the total aboveground biomass; -Bio indicates the aboveground biomass; R/S indicates the ratio of root to shoot biomass; -N indicates the concentration of total nitrogen; -P indicates the concentration of total phosphorus; -TN indicates the total nitrogen content; -TP indicates the total phosphorus content; PTN indicates the total nitrogen content of all aboveground biomass; PTP indicates the total phosphorus content of all aboveground biomass.

The RDA on soil microbial community constrained by soil properties was conducted to quantify the effects of soil variables on the variation in soil microbial composition (Figure 4A). The first two axes explained 67.30% of the variation in the soil microbial community composition. The concentrations of soil AN, AP, and pH were correlated to RDA1, especially AN and AP statistically significantly explained most variations. We then used Pearson’s correlations to decipher drivers for these significant decay relationships between microbes and soil properties. From individual PLFAs to functional populations, and then to microbial community levels, the soil microbes were consistently positively correlated with the concentration of SOM, AK, and AN, and negatively correlated with soil pH (Figure 4D).

The RDA was also used for plant community that was constrained by soil properties (Figure 4B). The first two axes explained 50.07% of the variation in plant community, and the concentration of SMC, NH_4_^+^-N, and AP could explain most variation. We further conducted the Pearson’s correlation analysis between soil properties and plants, and the results showed that the concentration of soil NH_4_^+^-N was significantly correlated with *Gramineae* biomass, and the total N and P contents of *Gramineae* (Figure 4C). In addition, the concentration of soil AP had a significant correlation with aboveground biomass (Figure 4C).

**FIGURE 5:**
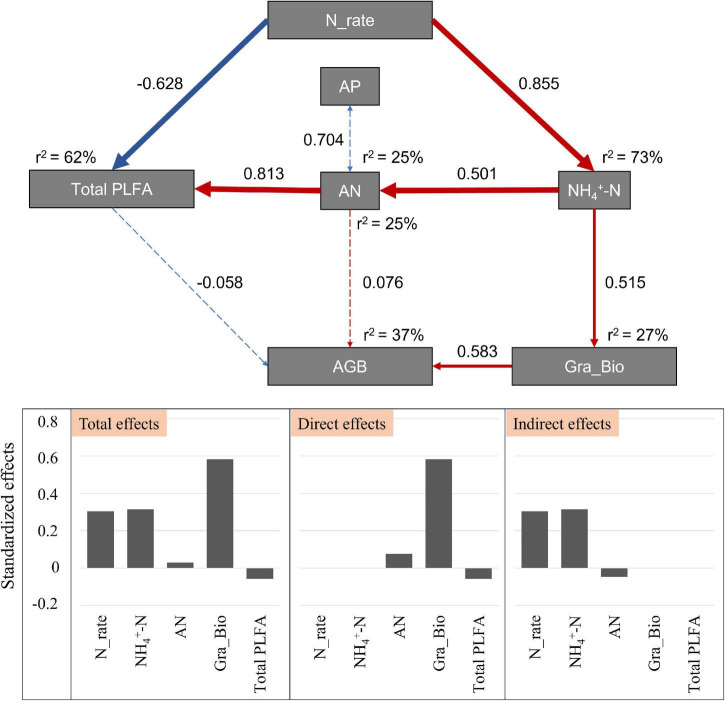
Effects of environmental variables and soil microbes on aboveground biomass after N addition by structural equation model. Blue solid arrows indicate significant positive relationships, and red solid arrows indicate significant negative relationships. Blue dotted arrows indicate negative relationships, and red dotted arrows indicate positive relationships. The thickness of the arrow represents the strength of the relationship. Numbers next to the pathway represent the standardized path coefficients. r2 represents the amount of interpretation. Bar graphs are the standardized effects from SEM on the aboveground biomass. AP indicates the soil available P; AN indicates the soil available N; AGB indicates the total aboveground biomass; Gra_Bio indicates the aboveground biomass of Gramineae.

The SEM was used to reveal the possible pathways through which soil and microbial attributes structure the aboveground biomass along the gradient of N application (χ^2^ = 14.714; *Df* = 12; *P* = 0.257; Figure 5). This model could explain 37% of the variance in aboveground biomass, and 62% of the variance in microbial biomass. N addition had direct negative effects on soil microbial biomass, and positive indirect effects *via* environmental variables (i.e., NH_4_^+^-N and AN). For the variation of aboveground biomass, *Gramineae* biomass explained the largest proportion (58.3%), and N addition and soil NH_4_^+^-N *via Gramineae* biomass explained 30.5 and 31.5% of the variation in the aboveground biomass, respectively (Figure 5).

## Discussion

Our results reveal that plants have limited responses to N addition in the Tibetan alpine steppe, while soil microbes remain unchanged. Under higher P conditions, the total biomass N content of plant community and *Gramineae* population, and the biomass of *Gramineae* showed linear trends with the increasing gradient of N addition. Conversely, soil microbes had no significant changes facing N addition from individual PLFAs to microbial community levels. These results confirm that plants, especially dominant population, have more responses to N addition compared to soil microbes in the Tibetan alpine steppe, which is consistent with recent findings in the Songnen grassland of China (Gao et al., 2019); and soil N is the limited element for plant growth, but not for soil microbes. These findings improve our understanding of the plant and microbes as indicators of soil quality (Schloter et al., 2003) and ecosystem services (Pommier et al., 2018).

These varied responses of soil microbes and plants to N addition are likely due to their different correlations with environmental variables. The microbial community was usually constructed by their surroundings (Delgado-Baquerizo et al., 2018; Wu et al., 2019). After applying fertilizers, urea was firstly hydrolyzed to NH_4_^+^, and then denitrified to NO_3_^–^ by ammonia-oxidizing bacteria (Dong et al., 2020a), resulting in more AN (ammonium + nitrite + nitrate) in the soil (Ma et al., 1999). However, only soil NH_4_^+^ was increased after N and P addition in this study, and no changes of soil AN was found, which may be due to the higher N loss in this area (Che et al., 2017), especially the higher preference of NO_3_^–^-N by the local dominant plants (*S*. *purpurea* and *L*. *leontopodioide*) than NH_4_^+^-N (Hong et al., 2017; Dong et al., 2020a), causing the nitrification product (i.e., NO_3_^–^) to be immediately absorbed by plants (Caffrey et al., 2007; Shen et al., 2008; Dong et al., 2020a). Importantly, soil AN, SOM, pH, and AK were the main factors for soil microbes, while these parameters remained unchanged. As a result, the soil microbes showed no responses to N addition.

The N demand of plants and their preference for different N forms structured their responses to N addition in this study. Plants have evolved many sophisticated strategies to support their nutrient acquisition and growth (Biemelt and Sonnewald, 2006). According to our results, some plant traits (e.g., the *Gramineae* biomass, the TN content of *Gramineae*, and the aboveground biomass) showed linear trends with increasing N rate, which highlighted the N limitation for plant growth and N acquisition in this study. Furthermore, the monocotyledonous species *S. purpurea* has higher N absorption rates than the dicotyledonous species *L. pusillum* (Liu et al., 2013; Hong et al., 2018), and thus most proportion of N was absorbed by *S. purpurea*, resulting in higher plant biomass production of *Gramineae*. Interestingly, the total P content of *Gramineae* also showed strong correlations with soil NH_4_^+^ and was co-enhanced by N addition, indicating the N:P balance for plant productivity and growth (Chen and Chen, 2021). In addition, the soil ammonium, nitrite, and nitrate make up the soil AN, and we found that plants and soil NH_4_^+^ had positive correlations, while AN remained unchanged, which implied the negative correlations between plants and AN except soil NH_4_^+^, highlighting that these plants prefer nitrate in the Tibetan alpine steppe.

Our findings showed that plants had more sensitive responses to N addition than soil microbes, highlighting the dominant roles of plant in plant–microbe interactions. Plants usually play central roles in complex food webs, with numerous organisms relying on their products of photosynthesis (Gruden et al., 2020). One of the most important ways was using root exudates to shape soil microbial community, but it was usually varied between different plant species or soil types (Kourtev et al., 2002; Haichar et al., 2008; Berg and Smalla, 2009). These findings must be based on their tight relationships. In this study, we found that N addition affected plants and soil microbes in different ways, with indirectly shifting plants *via* soil NH_4_^+^ and directly altering soil microbes. Compared to soil microbes, plants are a superior competitor for N uptake, and the fertilized N was immediately absorbed by plants to lessen their N limitation (Dong et al., 2020b). However, N addition reduced the correlations between plants and soil microbes (Wei et al., 2013), and simultaneously resulted in less photosynthate transport to soil surroundings from roots (Currey et al., 2011). In addition, under the ample P scenarios, we cannot figure out the P limitation for plants and soil microbes in this study, but our former study revealed that P was the limiting factor for soil microbes (Dong et al., 2020b). Taken together, plants absorbed the added-N immediately and the soil surroundings remained constant, resulting in the positive sensitive responses of plants while no changes for soil microbes.

We must point out that this study was conducted at a higher P level at the beginning of fertilization (after 2 years). These findings were limited, but we figured out the varied responses of plants and soil microbes to N addition, and the mechanisms of their different responses. We believe that our results can improve the prediction of responses of plants and microorganisms to N addition in the Tibetan alpine steppe, which might help us to find solutions to global climate changes we face. In addition, the responses of plants and soil microbes at different N rates and different P levels, and the long-term observation will be needed in the future to fully understand the stability of plants and soil microbes to nutrient addition in the Tibetan alpine steppe.

## Conclusion

In the Tibetan alpine steppe, N was the limiting factor for plants, especially for the dominant functional groups that were indicated by their biomass and N content. These positive responses were related to the soil AN except ammonium, including soil nitrite and nitrate. Soil microbes remained unchanged, which was due to the lessen relationships with plants and their lower competitiveness for N uptake than plants after N addition. We can conclude that N addition was first beneficial to the dominant plants, by increasing their production and nutrient acquisition and loosening their correlations with soil microbes. These findings would help us to select proper indicators to evaluate the soil quality and ecosystem services at the beginning after fertilization and to understand the plant–microbe interaction in the alpine steppe.

## Data Availability Statement

The original contributions presented in this study are included in the article/Supplementary Material, further inquiries can be directed to the corresponding author.

## Author Contributions

XC, HN, and SW designed the experiment. JD, JZ, CZ, and ZP conducted the experiment. JD and LL analyzed the data. All authors prepared and approved the manuscript.

## Conflict of Interest

The authors declare that the research was conducted in the absence of any commercial or financial relationships that could be construed as a potential conflict of interest.

## Publisher’s Note

All claims expressed in this article are solely those of the authors and do not necessarily represent those of their affiliated organizations, or those of the publisher, the editors and the reviewers. Any product that may be evaluated in this article, or claim that may be made by its manufacturer, is not guaranteed or endorsed by the publisher.

## Footnotes

1
www.r-project.org

## References

[B1] Adesemoye A. O. Torbert H. A. Kloepper J. W. (2009). Plant grow11th-promoting rhizobacteria allow reduced application rates of chemical fertilizers. *Microb. Ecol.* 58 921–929. 10.1007/s00248-009-9531-y 19466478

[B2] Adomako M. O. Xue W. Du D.-L. Yu F.-H. (2022). Soil microbe-mediated n:p stoichiometric effects on *Solidago canadensis* performance depend on nutrient levels. *Microb. Ecol.* 83 960–970. 10.1007/s00248-021-01814-8 34279696

[B3] Bao S. D. (2000). *Soil and Agricultural Chemistry Analysis*, 3rd Edn. Beijing: Chinese Agriculture Press.

[B4] Basto S. Thompson K. Phoenix G. Sloan V. Leake J. Rees M. (2015). Long-term nitrogen deposition depletes grassland seed banks. *Nat. Commun.* 6:6185. 10.1038/ncomms7185 25649868

[B5] Baumann F. He J.-S. Schmidt K. KÜHn P. Scholten T. (2009). Pedogenesis, permafrost, and soil moisture as controlling factors for soil nitrogen and carbon contents across the Tibetan Plateau. *Glob. Change Biol.* 15 3001–3017. 10.1111/j.1365-2486.2009.01953.x

[B6] Berg G. Smalla K. (2009). Plant species and soil type cooperatively shape the structure and function of microbial communities in the rhizosphere. *FEMS Microbiol. Ecol.* 68 1–13. 10.1111/j.1574-6941.2009.00654.x 19243436

[B7] Biemelt S. Sonnewald U. (2006). Plant–microbe interactions to probe regulation of plant carbon metabolism. *J. Plant Physiol.* 163 307–318. 10.1016/j.jplph.2005.10.011 16368160

[B8] Bird E. J. Choi Y. D. (2017). Response of native plants to elevated soil nitrogen in the sand dunes of Lake Michigan, USA. *Biol. Conserv.* 212 398–405. 10.1016/j.biocon.2016.12.001

[B9] Bobbink R. Hicks K. Galloway J. Spranger T. Alkemade R. Ashmore M. (2010). Global assessment of nitrogen deposition effects on terrestrial plant diversity: a synthesis. *Ecol. Appl.* 20 30–59. 10.1890/08-1140.1 20349829

[B10] Bobbink R. Hornung M. Roelofs J. G. M. (1998). The effects of air-borne nitrogen pollutants on species diversity in natural and semi-natural European vegetation. *J. Ecol.* 86 717–738. 10.1046/j.1365-2745.1998.8650717.x

[B11] Caffrey J. M. Bano N. Kalanetra K. Hollibaugh J. T. (2007). Ammonia oxidation and ammonia-oxidizing bacteria and archaea from estuaries with differing histories of hypoxia. *ISME J.* 1 660–662. 10.1038/ismej.2007.79 18043673

[B12] Che R. Wang F. Wang W. Zhang J. Zhao X. Rui Y. (2017). Increase in ammonia-oxidizing microbe abundance during degradation of alpine meadows may lead to greater soil nitrogen loss. *Biogeochemistry* 136 341–352. 10.1007/s10533-017-0399-5

[B13] Chen D. Xing W. Lan Z. Saleem M. Wu Y. Hu S. (2019). Direct and indirect effects of nitrogen enrichment on soil organisms and carbon and nitrogen mineralization in a semi-arid grassland. *Funct. Ecol.* 33 175–187. 10.1111/1365-2435.13226

[B14] Chen J.-B. Dong C.-C. Yao X.-D. Wang W. (2018). Effects of nitrogen addition on plant biomass and tissue elemental content in different degradation stages of temperate steppe in northern China. *J. Plant Ecol.* 11 730–739. 10.1093/jpe/rtx035

[B15] Chen X. Chen H. Y. H. (2021). Plant mixture balances terrestrial ecosystem C:N:P stoichiometry. *Nat. Commun.* 12:4562. 10.1038/s41467-021-24889-w 34315908 PMC8316448

[B16] Cordell D. Drangert J.-O. White S. (2009). The story of phosphorus: global food security and food for thought. *Glob. Environ. Change* 19 292–305. 10.1016/j.gloenvcha.2008.10.009

[B17] Currey P. M. Johnson D. Dawson L. A. van der Wal R. Thornton B. Sheppard L. J. (2011). Five years of simulated atmospheric nitrogen deposition have only subtle effects on the fate of newly synthesized carbon in *Calluna vulgaris* and *Eriophorum vaginatum*. *Soil Biol. Biochem.* 43 495–502. 10.1016/j.soilbio.2010.11.003

[B18] Delgado-Baquerizo M. Oliverio Angela M. Brewer Tess E. Benavent-González A. Eldridge David J. Bardgett Richard D. (2018). A global atlas of the dominant bacteria found in soil. *Science* 359 320–325. 10.1126/science.aap9516 29348236

[B19] Dong J. Che R. Jia S. Wang F. Zhang B. Cui X. (2020a). Responses of ammonia-oxidizing archaea and bacteria to nitrogen and phosphorus amendments in an alpine steppe. *Eur. J. Soil Sci.* 71 940–954. 10.1111/ejss.12911

[B20] Dong J. Wang S. Niu H. Cui X. Li L. Pang Z. (2020b). Responses of soil microbes and their interactions with plant community after nitrogen and phosphorus addition in a Tibetan alpine steppe. *J. Soils Sediments* 20 2236–2247. 10.1007/s11368-020-02586-3

[B21] Dong J. Cui X. Wang S. Wang F. Pang Z. Xu N. (2016). Changes in biomass and quality of alpine steppe in response to N & P fertilization in the Tibetan Plateau. *PLoS One* 11:e0156146. 10.1371/journal.pone.0156146 27223104 PMC4880335

[B22] Eghball B. (2002). Soil properties as influenced by phosphorus- and nitrogen-based manure and compost applications. *Agron. J.* 94 128–135. 10.2134/agronj2002.1280

[B23] Elser J. Bennett E. (2011). A broken biogeochemical cycle. *Nature* 478 29–31. 10.1038/478029a 21979027

[B24] Foster B. L. Gross K. L. (1998). Species richness in a successional grassland: effects of nitrogen enrichment and plant litter. *Ecology* 79 2593–2602. 10.1890/0012-9658(1998)079[2593:SRIASG]2.0.CO;2

[B25] Frey S. D. Knorr M. Parrent J. L. Simpson R. T. (2004). Chronic nitrogen enrichment affects the structure and function of the soil microbial community in temperate hardwood and pine forests. *For. Ecol. Manag.* 196 159–171. 10.1016/j.foreco.2004.03.018

[B26] Frostegård A. Bååth E. (1996). The use of phospholipid fatty acid analysis to estimate bacterial and fungal biomass in soil. *Biol. Fertil. Soils* 22 59–65. 10.1007/BF00384433

[B27] Fu G. Shen Z.-X. (2016). Response of alpine plants to nitrogen addition on the Tibetan Plateau: a meta-analysis. *J. Plant Growth Regul.* 35 974–979. 10.1007/s00344-016-9595-0

[B28] Galloway J. N. Dentener F. J. Capone D. G. Boyer E. W. Howarth R. W. Seitzinger S. P. (2004). Nitrogen cycles: past, present, and future. *Biogeochemistry* 70 153–226. 10.1007/s10533-004-0370-0

[B29] Gao Y. Sun S. Xing F. Mu X. Bai Y. (2019). Nitrogen addition interacted with salinity-alkalinity to modify plant diversity, microbial PLFAs and soil coupled elements: a 5-year experiment. *Appl. Soil Ecol.* 137 78–86. 10.1016/j.apsoil.2019.01.011

[B30] Gruden K. Lidoy J. Petek M. Podpeèan V. Flors V. Papadopoulou K. K. (2020). Ménage à trois: unraveling the mechanisms regulating plant–microbe–arthropod interactions. *Trends Plant Sci.* 25 1215–1226. 10.1016/j.tplants.2020.07.008 32828689

[B31] Haichar F. E. Z. Marol C. Berge O. Rangel-Castro J. I Prosser J. I Balesdent J. (2008). Plant host habitat and root exudates shape soil bacterial community structure. *ISME J.* 2 1221–1230. 10.1038/ismej.2008.80 18754043

[B32] Han Y. Feng G. Swaney D. P. Dentener F. Koeble R. Ouyang Y. (2020). Global and regional estimation of net anthropogenic nitrogen inputs (NANI). *Geoderma* 361:114066. 10.1016/j.geoderma.2019.114066

[B33] Hong J. Ma X. Yan Y. Zhang X. Wang X. (2018). Which root traits determine nitrogen uptake by alpine plant species on the Tibetan Plateau? *Plant Soil* 424 63–72. 10.1007/s11104-017-3434-3

[B34] Hong J. Ma X. Zhang X. Wang X. (2017). Nitrogen uptake pattern of herbaceous plants: coping strategies in altered neighbor species. *Biol. Fertil. Soils* 53 729–735. 10.1007/s00374-017-1230-0

[B35] Hu Y.-L. Zeng D.-H. Liu Y.-X. Zhang Y.-L. Chen Z.-H. Wang Z.-Q. (2010). Responses of soil chemical and biological properties to nitrogen addition in a *Dahurian larch* plantation in Northeast China. *Plant Soil* 333 81–92. 10.1007/s11104-010-0321-6

[B36] Kang H. Gao H. Yu W. Yi Y. Wang Y. Ning M. (2018). Changes in soil microbial community structure and function after afforestation depend on species and age: case study in a subtropical alluvial island. *Sci. Total Environ.* 625 1423–1432. 10.1016/j.scitotenv.2017.12.180 29996439

[B37] Kourtev P. S. Ehrenfeld J. G. Häggblom M. (2002). Exotic plant species alter the microbial community structure and function in the soil. *Ecology* 83 3152–3166. 10.1890/0012-9658(2002)083[3152:EPSATM]2.0.CO;2

[B38] Leff J. W. Jones S. E. Prober S. M. Barberán A. Borer E. T. Firn J. L. (2015). Consistent responses of soil microbial communities to elevated nutrient inputs in grasslands across the globe. *Proc. Natl. Acad. Sci. U.S.A* 112 10967–10972. 10.1073/pnas.1508382112 26283343 PMC4568213

[B39] Liao C. F. H. (1981). Devarda’s alloy method for total nitrogen determination. *Soil Sci. Soc. Am. J.* 45 852–855. 10.2136/sssaj1981.03615995004500050005x

[B40] Liu Y. Xu R. Xu X. Wei D. Wang Y. Wang Y. (2013). Plant and soil responses of an alpine steppe on the Tibetan Plateau to multi-level nitrogen addition. *Plant Soil* 373 515–529. 10.1007/s11104-013-1814-x

[B41] Lu X. Mo J. Gilliam F. S. Zhou G. Fang Y. (2010). Effects of experimental nitrogen additions on plant diversity in an old-growth tropical forest. *Glob. Change Biol.* 16 2688–2700. 10.1111/j.1365-2486.2010.02174.x

[B42] Lü X.-T. Reed S. Yu Q. He N.-P. Wang Z.-W. Han X.-G. (2013). Convergent responses of nitrogen and phosphorus resorption to nitrogen inputs in a semiarid grassland. *Glob. Change Biol.* 19 2775–2784. 10.1111/gcb.12235 23625746

[B43] Lü X.-T. Reed S. C. Yu Q. Han X.-G. (2016). Nutrient resorption helps drive intra-specific coupling of foliar nitrogen and phosphorus under nutrient-enriched conditions. *Plant Soil* 398 111–120. 10.1007/s11104-015-2642-y

[B44] Luo R. Fan J. Wang W. Luo J. Kuzyakov Y. He J.-S. (2019). Nitrogen and phosphorus enrichment accelerates soil organic carbon loss in alpine grassland on the Qinghai-Tibetan Plateau. *Sci. Total Environ.* 650 303–312. 10.1016/j.scitotenv.2018.09.038 30199676

[B45] Ma B. Zhou X. Zhang Q. Qin M. Hu L. Yang K. (2019). How do soil micro-organisms respond to N, P and NP additions? Application of the ecological framework of (co-)limitation by multiple resources. *J. Ecol.* 107 2329–2345. 10.1111/1365-2745.13179

[B46] Ma X. Zhang Q. Zheng M. Gao Y. Yuan T. Hale L. (2019). Microbial functional traits are sensitive indicators of mild disturbance by lamb grazing. *ISME J.* 13 1370–1373. 10.1038/s41396-019-0354-7 30700789 PMC6474220

[B47] Ma B. L. Dwyer L. M. Gregorich E. G. (1999). Soil nitrogen amendment effects on seasonal nitrogen mineralization and nitrogen cycling in maize production. *Agron. J.* 91 1003–1009. 10.2134/agronj1999.9161003x

[B48] Mu X. Chen Y. (2021). The physiological response of photosynthesis to nitrogen deficiency. *Plant Physiol. Biochem.* 158 76–82. 10.1016/j.plaphy.2020.11.019 33296848

[B49] Orwin K. H. Dickie I. A. Holdaway R. Wood J. R. (2018). A comparison of the ability of PLFA and 16S rRNA gene metabarcoding to resolve soil community change and predict ecosystem functions. *Soil Biol. Biochem.* 117 27–35. 10.1016/j.soilbio.2017.10.036

[B50] Peng Y. Peng Z. Zeng X. Houx J. H. (2019). Effects of nitrogen-phosphorus imbalance on plant biomass production: a global perspective. *Plant Soil* 436 245–252. 10.1007/s11104-018-03927-5

[B51] Peñuelas J. Poulter B. Sardans J. Ciais P. van der Velde M. Bopp L. (2013). Human-induced nitrogen–phosphorus imbalances alter natural and managed ecosystems across the globe. *Nat. Commun.* 4:2934. 10.1038/ncomms3934 24343268

[B52] Pommier T. Cantarel A. A. M. Grigulis K. Lavorel S. Legay N. Baxendale C. (2018). The added value of including key microbial traits to determine nitrogen-related ecosystem services in managed grasslands. *J. Appl. Ecol.* 55 49–58. 10.1111/1365-2664.13010

[B53] Rashid M. I. Mujawar L. H. Shahzad T. Almeelbi T. Ismail I. M. I. Oves M. (2016). Bacteria and fungi can contribute to nutrients bioavailability and aggregate formation in degraded soils. *Microbiol. Res.* 183 26–41. 10.1016/j.micres.2015.11.007 26805616

[B54] Richardson A. E. Barea J.-M. McNeill A. M. Prigent-Combaret C. (2009). Acquisition of phosphorus and nitrogen in the rhizosphere and plant growth promotion by microorganisms. *Plant Soil* 321 305–339. 10.1007/s11104-009-9895-2

[B55] Sarathchandra S. U. Ghani A. Yeates G. W. Burch G. Cox N. R. (2001). Effect of nitrogen and phosphate fertilisers on microbial and nematode diversity in pasture soils. *Soil Biol. Biochem.* 33 953–964. 10.1016/S0038-0717(00)00245-5

[B56] Schloter M. Dilly O. Munch J. C. (2003). Indicators for evaluating soil quality. *Agric. Ecosyst. Environ.* 98 255–262. 10.1016/S0167-8809(03)00085-9

[B57] Shen J.-P. Zhang L.-M. Zhu Y.-G. Zhang J.-B. He J.-Z. (2008). Abundance and composition of ammonia-oxidizing bacteria and ammonia-oxidizing archaea communities of an alkaline sandy loam. *Environ. Microbiol.* 10 1601–1611. 10.1111/j.1462-2920.2008.01578.x 18336563

[B58] Simkin S. M. Allen E. B. Bowman W. D. Clark C. M. Belnap J. Brooks M. L. (2016). Conditional vulnerability of plant diversity to atmospheric nitrogen deposition across the United States. *Proc. Natl. Acad. Sci. U.S.A.* 113 4086–4091. 10.1073/pnas.1515241113 27035943 PMC4839424

[B59] Soons M. B. Hefting M. M. Dorland E. Lamers L. P. M. Versteeg C. Bobbink R. (2017). Nitrogen effects on plant species richness in herbaceous communities are more widespread and stronger than those of phosphorus. *Biol. Conserv.* 212 390–397. 10.1016/j.biocon.2016.12.006

[B60] Stevens C. J. David T. I. Storkey J. (2018). Atmospheric nitrogen deposition in terrestrial ecosystems: its impact on plant communities and consequences across trophic levels. *Funct. Ecol.* 32 1757–1769. 10.1111/1365-2435.13063

[B61] Stevens C. J. Dise N. B. Mountford J. O. Gowing D. J. (2004). Impact of nitrogen deposition on the species richness of grasslands. *Science* 303 1876–1879. 10.1126/science.1094678 15031507

[B62] Su J.-Q. Ding L.-J. Xue K. Yao H.-Y. Quensen J. Bai S.-J. (2015). Long-term balanced fertilization increases the soil microbial functional diversity in a phosphorus-limited paddy soil. *Mol. Ecol.* 24 136–150. 10.1111/mec.13010 25410123

[B63] Tessier J. T. Raynal D. J. (2003). Use of nitrogen to phosphorus ratios in plant tissue as an indicator of nutrient limitation and nitrogen saturation. *J. Appl. Ecol.* 40 523–534. 10.1046/j.1365-2664.2003.00820.x

[B64] Treseder K. K. (2008). Nitrogen additions and microbial biomass: a meta-analysis of ecosystem studies. *Ecol. Lett.* 11 1111–1120. 10.1111/j.1461-0248.2008.01230.x 18673384

[B65] Van Der Heijden M. G. A. Bardgett R. D. Van Straalen N. M. (2008). The unseen majority: soil microbes as drivers of plant diversity and productivity in terrestrial ecosystems. *Ecol. Lett.* 11 296–310. 10.1111/j.1461-0248.2007.01139.x 18047587

[B66] Vitousek P. M. Porder S. Houlton B. Z. Chadwick O. A. (2010). Terrestrial phosphorus limitation: mechanisms, implications, and nitrogen–phosphorus interactions. *Ecol. Appl.* 20 5–15. 10.1890/08-0127.1 20349827

[B67] Wei C. Yu Q. Bai E. Lü X. Li Q. Xia J. (2013). Nitrogen deposition weakens plant–microbe interactions in grassland ecosystems. *Glob. Change Biol.* 19 3688–3697. 10.1111/gcb.12348 23925948

[B68] Wu L. Ning D. Zhang B. Li Y. Zhang P. Shan X. (2019). Global diversity and biogeography of bacterial communities in wastewater treatment plants. *Nat. Microbiol.* 4 1183–1195. 10.1038/s41564-019-0426-5 31086312

[B69] Xiao D. Xiao S. Ye Y. Zhang W. He X. Wang K. (2019). Microbial biomass, metabolic functional diversity, and activity are affected differently by tillage disturbance and maize planting in a typical karst calcareous soil. *J. Soils Sediments* 19 809–821. 10.1007/s11368-018-2101-5

[B70] Xu W. Luo X. S. Pan Y. P. Zhang L. Tang A. H. Shen J. L. (2015). Quantifying atmospheric nitrogen deposition through a nationwide monitoring network across China. *Atmos. Chem. Phys.* 15 12345–12360. 10.5194/acp-15-12345-2015

[B71] Zeng J. Liu X. J. Song L. Lin X. G. Zhang H. Y. Shen C. C. (2016). Nitrogen fertilization directly affects soil bacterial diversity and indirectly affects bacterial community composition. *Soil Biol. Biochem.* 92 41–49. 10.1016/j.soilbio.2015.09.018

[B72] Zhong L. Ma Y. Xue Y. Piao S. (2019). Climate change trends and impacts on vegetation greening over the Tibetan Plateau. *J. Geophys. Res. Atmos.* 124 7540–7552. 10.1029/2019JD030481

[B73] Zosso C. U. Wiesenberg G. L. B. (2021). Methylation procedures affect PLFA results more than selected extraction parameters. *J. Microbiol. Methods* 182:106164. 10.1016/j.mimet.2021.106164 33582123

