# Supplementary material for: Effects of Nitrogen Addition on Plant Properties and Microbiomes Under High Phosphorus Addition Level in the Alpine Steppe
Source: Front Plant Sci. 2022 Jun 20;13:894365. doi: 10.3389/fpls.2022.894365 (PMC9251499; doi:10.3389/fpls.2022.894365)
Supplement: Supplementary file 1 [file Data_Sheet_1.docx]

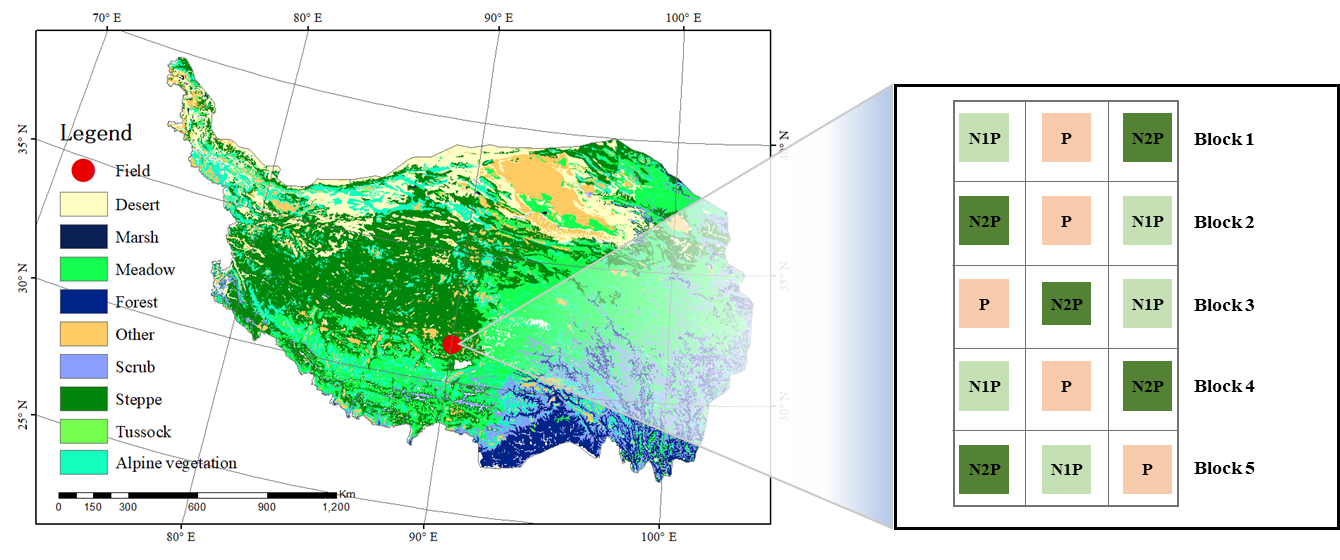


Figure S1 The field location and the layout map of design. P indicates only phosphorus addition, N1P indicates 7.5 g.N.m^-2^.yr^-1^ addition with P addition, N2P indicates 15 g.N.m^-2^.yr^-1^ addition with P addition.


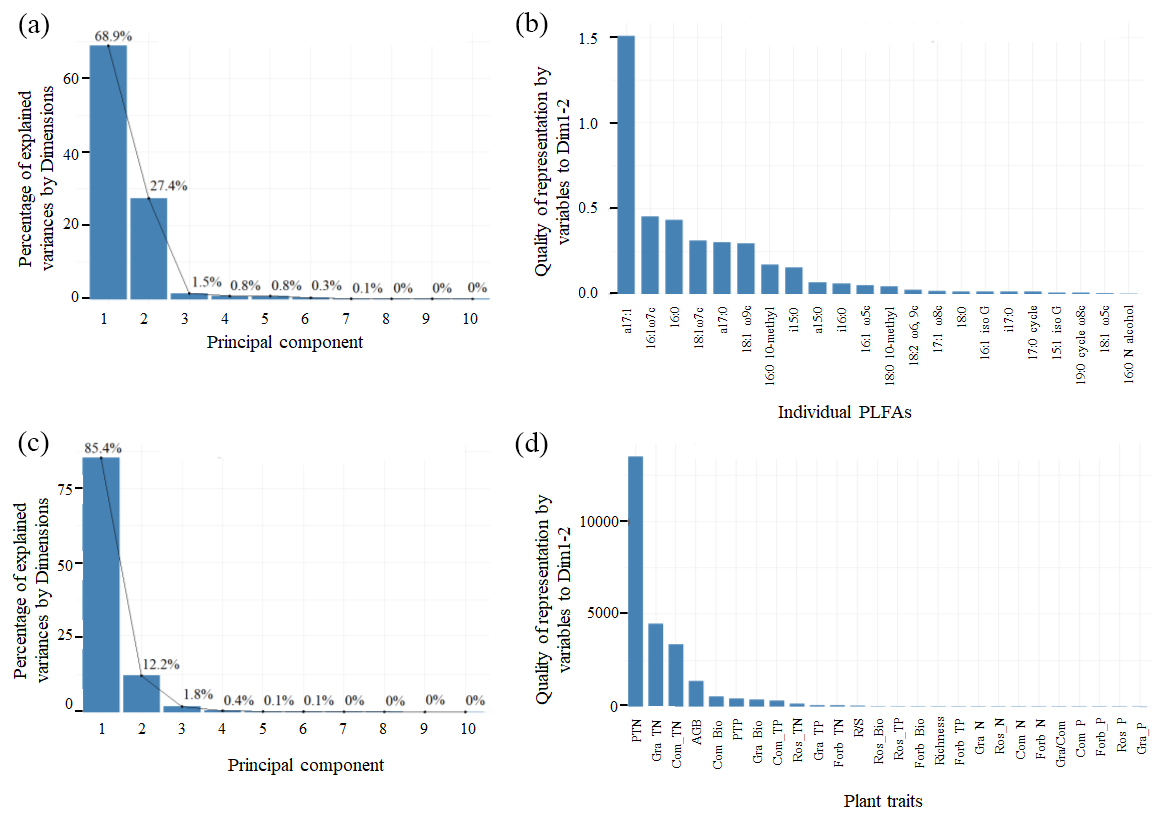


Figure S2 Principal component analysis (PCA) of microbial community and plant variables. (a) is the percentage of each principal component (PC) explained the variances for the 10 variances of soil microbial community; (b) is the quality of representation of variables to PC1 and PC2 of soil microbial community; (c) is the percentage of PC explained the variances for the 10 variances of plants; and (d) is the quality of representation of variables to PC1 and PC2 of plants. *Gra* indicates *Gramineae*; *Com* indicates *Compositae*; Cyp indicates *Cypositae*; AGB indicates the total aboveground biomass; -Bio indicates the aboveground biomass; R/S indicates the ratio of root to shoot biomass; Gra/Com indicates the ratio of *Gramineae* to *Compositae* biomass; -N indicates the concentration of total nitrogen; -P indicates the concentration of total phosphorus; -TN indicates the total nitrogen content; -TP indicates the total phosphorus content; PTN indicates the total nitrogen content of all aboveground biomass; PTP indicates the total phosphorus content of all aboveground biomass.

Table S1 The changes of soil properties (mean ± SE) after N addition. Different letters indicate significant differences between N application rates (n=4, *P* < 0.05).

| N rate  Properties | 0 g.N.m^-2^.yr^-1^ | 7.5 g.N.m^-2^.yr^-1^ | 15 g.N.m^-2^.yr^-1^ |
| --- | --- | --- | --- |
| SMC | 0.1225 ± 0.0085 a | 0.1275 ± 0.0085 a | 0.1200 ± 0.0091 a |
| **NH_4_^+^-N** | **4.1525 ± 0.9210 b** | **11.5950 ± 2.2382 b** | **28.0675 ± 4.9777 a** |
| SOM | 24.7175 ± 2.5278 a | 26.7775 ± 1.3304 a | 24.2900 ± 1.0277 a |
| AP | 42.6075 ± 7.6781 a | 53.7025 ± 5.9372 a | 39.7475 ± 6.9826 a |
| AK | 183.9800 ± 20.4513 a | 190.1550 ± 14.0516 a | 186.9800 ± 9.7644 a |
| TN | 1.6100 ± 0.1332 a | 1.5825 ± 0.0833 a | 1.5350 ± 0.1093 a |
| AN | 122.0025 ± 10.5659 a | 139.5450 ± 6.1193 a | 140.8225 ± 12.6321 a |
| TP | 0.4625 ± 0.0325 a | 0.4825 ± 0.0275 a | 0.4475 ± 0.0263 a |
| pH | 7.2300 ± 0.1116 a | 7.1400 ± 0.0540 a | 7.1200 ± 0.0878 a |

Note: SMC indicates the soil moisture content; SOM indicates the soil organic matter content; AP indicates the soil available P; AK indicates the soil available potassium; TN indicates the soil total N content; AN indicates the soil available N; TP indicates the soil total P content.
